# A Minimal Anaphase Promoting Complex/Cyclosome (APC/C) in *Trypanosoma brucei*

**DOI:** 10.1371/journal.pone.0059258

**Published:** 2013-03-22

**Authors:** Mohamed Bessat, Giselle Knudsen, Alma L. Burlingame, Ching C. Wang

**Affiliations:** Department of Pharmaceutical Chemistry, University of California San Francisco, San Francisco, California, United States of America; University of Texas Medical School at Houston, United States of America

## Abstract

The anaphase-promoting complex/cyclosome (APC/C) is a multi-subunit E3 ubiquitin ligase that initiates chromosome segregation and mitotic exit by targeting critical cell-cycle regulators for proteolytic destruction. Previously, seven APC/C subunit homologues were identified in the genome of *Trypanosoma brucei.* In the present study, we tested five of them in yeast complementation studies and found none of them capable of complementing the yeast mutants lacking the corresponding subunits, suggesting significant discrepancies between the two APC/C’s. Subunit homologues of mitotic checkpoint complex (MCC) have not yet been identified in T. brucei, raising the possibility that a MCC-APC/C complex equivalent may not exist in T. brucei. We performed tandem affinity purification of the protein complex containing a APC1 fusion protein expressed in the cells enriched in different phases of the cell cycle of procyclic form T. brucei, and compared their protein profiles using LC-MS/MS analyses. The seven putative APC/C subunits were identified in the protein complex throughout the cell cycle together with three additional proteins designated the associated proteins (AP) AP1, AP2 and AP3. Abundance of the 10 proteins remained relatively unchanged throughout the cell cycle, suggesting that they are the core subunits of APC/C. AP1 turned out to be a homologue of APC4. An RNAi knockdown of APC4 and AP3 showed no detectable cellular phenotype, whereas an AP2 knockdown enriched the cells in G2/M phase. The AP2-depleted cells showed stabilized mitotic cyclin B. An accumulation of poly-ubiquitinated cyclin B was indicated in the cells treated with the proteasome inhibitor MG132, demonstrating the involvement of proteasome in degrading poly-ubiquitinated cyclin B. In all, a 10-subunit APC/C machinery with a conserved function is identified in T. brucei without linking to a MCC-like complex, thus indicating a unique T. brucei APC/C.

## Introduction

*Trypanosoma brucei*, is an early divergent protozoan parasite that causes African sleeping sickness in human and nagana in livestock. It has a complex biphasic life cycle that allows the cells to multiply in both the mammalian host and the insect vector tsetse flies [1]. There are many unique features in the cell cycle progression in *T. brucei* when compared with that in metazoa [2], [3]. For instance, cytokinesis in the bloodstream-form T. brucei is controlled by mitosis whereas that in the insect (procyclic) form is driven primarily by the duplication and segregation of basal bodies and its associated mitochondrial DNA complexes, the kinetoplasts [4], [5], [6]. Therefore, procyclic form cells can undergo cytokinesis in the absence of mitosis, whereas a mitotic arrest in bloodstream form cells inhibits cytokinesis with continued kinetoplast replication and segregation and nuclear DNA synthesis [2], [5], [6], which implicates fundamental differences in cell cycle controls between different life cycle forms of *T. brucei* and potential absence of the key cell cycle checkpoints. Cell division in both forms of T. brucei proceeds longitudinally along the dorsal line from the anterior to the posterior end of the cell [7], which contrasts significantly from that in metazoa [8], [9]. The mechanism of this distinctive form of cell division in *T. brucei* is initiated by a trans-localization of the chromosome passenger complex (CPC) from the midzone of central spindle across the nuclear envelope to the flagellar attachment zone (FAZ) during the final stage of mitosis [10]. The CPC then moves along the FAZ to the anterior end of the dividing cell and slides back toward the posterior end along the FAZ in an unzipping action to separate the dividing mother from the daughter [10]. The cell cycle of T. brucei has thus become one of the most intriguing subjects for further investigation in recent years.

The progression from metaphase to anaphase during mitosis of T. brucei appears, however, somewhat similar to that observed in other eukaryotes [2]. The chromosomes in the nucleus of T. brucei are replicated during S-phase and attached to the mitotic spindle and aligned in two closely associated parallel rows during metaphase [11]. The chromosomal duplexes are then pulled apart by the mitotic spindle into two separate entities during anaphase [11]. Metaphase-anaphase transition and mitotic exit in metazoa and yeast are controlled primarily by the anaphase promoting complex/cyclosome (APC/C) regulated by periodic association and dissociation with the mitotic checkpoint complex (MCC). APC/C is a multi-subunit E3 ubiquitin ligase consisting of 13 core subunit proteins in yeast that is inactivated by association with the effector proteins of the MCC complex in the prometaphase [12], [13], [14], [15], [16]. When proper alignment and attachment of the duplicated chromosomes to the mitotic spindle are achieved toward the end of metaphase, MCC and APC/C are dissociated from each other leaving a single MCC subunit protein, CDC20, with the APC/C to activate the latter to poly-ubiquitinate securin/Pds1 for 26S proteasomal degradation [17], [18], [19]. Securin/Pds1 inhibits the activity of a protease separase by binding to the latter during metaphase. Upon its destruction, separase is freed and activated and degrades the cohesin protein binding the sister chromatids for successful chromosome segregation and cell cycle progression to the anaphase [20]. Toward the late mitotic phase, a mitotic cyclin B is targeted by the activated APC/C for poly-ubiquitination and subsequent 26S proteasome-mediated degradation thus lowers the activity of cyclin-dependent kinas 1 (CDK1) for mitotic exit [21], [22].

In our previous study, we identified seven APC/C subunit homologues in T. brucei that include APC1, APC2, APC10/DOC1, APC11, CDC16, CDC23 and CDC27 [23]. There is also a CDC20 and a separase homologue found in T. brucei genome database (unpublished data and [24]). But no homologue of securin/Pds1 or any of the MCC subunit proteins has yet been identified in this organism. The separase homologue demonstrated a conserved role in catalyzing chromosome segregation in *T. brucei* [24], but an RNAi knockdown of CDC20 in procyclic form T. brucei showed no apparent mitotic arrest (unpublished data). Further RNAi knockdowns of each of the seven subunit proteins in the apparent APC/C of T. brucei showed that only depletion of APC1 or CDC27 resulted in mitotic arrest in both procyclic and bloodstream form cells [23], whereas knockdowns of the rest of the 5 subunits showed no apparent phenotype [23]. This outcome is similar to that from Saccharomyces cerevisiae, in which mutants of APC9 [25], MND2, SWM1 [26] or CDC26 [27] did not register any phenotype and were classified as the nonessential subunits of the APC/C in yeast.

There is thus a likely presence of functional APC/C in T. brucei [23]. But the previous results did not verify whether the 7 subunits constitute the entire core of a APC/C structure or if more protein subunits are involved in constituting the complex. Nor is it clear if the APC/C function is regulated by associating with a functional MCC-like complex or whether a functional homologue of securin/Pds1 and a functional homologue of CDC20 may exist and play pivotal roles in metaphase-anaphase transition in T. brucei.

In an effort to clarify these issues, we first used the method of yeast complementation to examine if any of the putative T. brucei APC/C subunits are capable of replacing those in S. cerevisiae, and found that among those tested, none was capable of substituting the yeast counterpart. We then used tandem affinity purification (TAP) and mass spectrometry to identify the composition of T. brucei APC/C and examined its potential association-dissociation with other proteins during the metaphase-anaphase transition. The outcome indicated that, throughout the entire cell cycle, there was a constant presence of an apparent APC/C complex consisting of 10 subunit proteins. Neither the CDC20 homologue nor any other protein was found detectable with the APC/C at any phase of the cell cycle, suggesting a unique mechanism of APC/C regulation in *T. brucei*. The mitotic cyclin B of *T. brucei* (cycB2/cyc6) [28] was, however, found poly-ubiquitinated by the APC/C and degraded by proteasome for mitotic exit as in other eukaryotes.

## Materials and Methods

### Yeast Complementation Assay

Temperature-sensitive (ts) S. cerevisiae mutants cdc16-1, cdc23-1, cdc27-1 and apc1-1 were kindly provided by Dr. D. Toczyski of UCSF [29]. The apc10-1 ts mutant was purchased from Open Biosystems (Thermo Scientific). A wild-type 303wt strain of S. cerevisiae was from Dr. P. Walter of UCSF. The genetic background among the yeast strains are all of Mat/a except for the apc10-1 mutant and 303 wt strain, which are of Mat/α. For the standard yeast propagation, cells were grown in Yeast extract/Peptone/Dextrose (YPD) medium. Full-length open reading frames (ORFs) of genes encoding CDC16, CDC23, CDC27, APC1 and APC10/DOC1 were amplified from the yeast and the *T. brucei* genomic DNAs by PCR using gene specific primers with restriction sites for cloning into the yeast expression plasmid, pRS416-ADH [30]. Individual constructs were introduced into the corresponding yeast mutants and expressed under the control of yeast specific ADH promoter [30]. The transformed cells were selected on the synthetic-defined (SD) minimal selection medium supplemented with the Drop-Out (DO) supplement–Ura (Clontech). Yeast transformation and selection were by the protocol of Gietz [31], [32]. Replica plates of transformed cells were incubated for 3 days at either the permissive (25°C) or the restrictive (37°C) temperature for indication of genetic complementation by the cloned genes.

### Trypanosome Cell Culture

Procyclic-form *T. brucei* cells of the wild type strain 427 and the modified strain 29-13 for RNAi studies [33] were both cultivated at 26°C in the Cunningham medium supplemented with 10% fetal bovine serum (Hyclone. Thermo scientific, USA). For the 29-13 strain, 15 µg ml^−1^ G418 and 50 µg ml^−1^ hygromycin B were added to the culture medium to maintain expression of T7 RNA polymerase and tetracycline repressor, respectively [33]. Cell densities were maintained at the mid-log phase of ∼5×10^6^ cells ml^−1^ through regular subculturings. Transfection and selection of the procyclic *T. brucei* cells were carried out as described previously [34].

### Expression of Protein A-tobacco Etch Virus (TEV) Protease Site-protein C Epitope (PTP)-tagged APC1 and Purification of the APC1-associated Protein Complex

The 3′-terminal fragment of T. brucei APC1 gene encompassing nucleotide sequence #4,668–#5,510 was amplified by PCR from the genomic DNA of the cells of 427 strain and cloned in frame into the pC-PTP-NEO plasmid to produce the pC-APC1-PTP construct [35]. The plasmid was linearized with XcmI and transfected into the strain 427 cells by electroporation for genome integration through homologous recombination. The transfected cells were selected under 40 µg ml^−1^ G418. Correct PTP tagging of the endogenous APC1 gene in the transfected cells was subsequently confirmed with PCR and DNA-sequencing. The transfected cell cultures were further diluted and distributed to 24-well plates at a calculated average of a single cell per well for further cell growth to identify the cloned cell line [36]. The cells showing consistent and optimal growth rates were selected and tested for expression of the PTP-tagged APC1 protein in Western blotting (see below) and stored in liquid nitrogen.

For purification of the PTP-APC1-containing protein complex from the cloned transfected cells, crude lysate was prepared from ∼1×10^9^ cells by sonication, and cleared by a brief centrifugation [35]. The cell lysate was then subjected to stepwise purifications through immunoglobulin G (IgG) column binding, TEV protease elution, protein C antibody column binding and EGTA chelating elution according to the established procedure [35]. Each step of the purification was monitored with Western blotting and SDS-PAGE stained with SYPRO Ruby (Invitrogen, CA). Individual protein bands in the final purified sample fractionated in SDS-PAGE and stained with SYPRO Ruby were sliced from the gel, diced and processed for in-gel trypsin digestion by a standard protocol (The UCSF Mass Spectrometry In-gel digestion procedure @ ms-facility.ucsf.edu/ingel.html) followed by mass spectrometric analysis (see below).

### Synchronization of the Cell Cycle Progression in *T. brucei*

Mid-log phase procyclic-form T. brucei cells expressing APC1-PTP were treated with 0.3 mM of hydroxyurea and incubated at 26°C for 16 hr to become synchronized in the late S phase [37]. Hydroxyurea was then washed off, and the late S phase cells were cultivated for synchronized cell cycle progression for 2.5 additional hrs for a cell population highly enriched in metaphase, and 4.5 hrs to enrich the cells in anaphase [10]. The unsynchronized cells are enriched in G1 phase. They and the other cell populations enriched in S phase, metaphase and anaphase were each used for the TAP purification of APC1-associated protein complexes and for comparisons of their protein profiles in SDS-PAGE-SYPRO Ruby gels and mass spectrometric analysis.

### Liquid Chromatography-tandem Mass Spectrometry (LC-MS/MS)

Following in-gel trypsin digestions, samples from individual protein bands were each analyzed using an LTQ-Orbitrap XL (Thermo) mass spectrometer. It was equipped with a 10,000 psi system nanoACUITY (Waters) UPLC instrument for peptide separation by reversed phase chromatography with a C18 column (BEH130, 1.7 µm bead size, 100 µm×100 mm). The LC was operated at a flow rate of 600 nL/min, and the peptides were separated using a linear gradient of acetonitrile from 2% to 30% in solvent A (0.1% formic acid in water) and solvent B (0.1% formic acid in 70% acetonitrile) over a period of 42 min. Survey scans were recorded over 310-1600 m/z range, and MS/MS was performed in data dependent acquisition mode with CID fragmentation on the six most intense precursor ions, measured in the ion trap.

Lists of mass spectrometric peaks were generated using the in-house software PAVA, and data were searched using the Protein Prospector software v. 5.10.0 [38]. Database searches were performed against the SwissProt database (downloaded March 21, 2012) to which was added the *T. brucei* subset of the TriTrypDB database v. 4.1 (downloaded June 21, 2012) totaling 563,498 entries. For estimation of false discovery rates, the database was concatenated with a fully randomized set of sequence entries [39]. Data were searched with mass tolerances of 20 ppm for parent and 0.6 Da for fragment ions.

For database searching, peptide sequences were matched as tryptic peptides with no missed cleavages, and carbamidomethylated cysteines as a fixed modification. Variable modifications included oxidation of methionine, N-terminal pyroglutamate from glutamine, loss of methionine and N-terminal acetylation. Protein Prospector score parameters were set at a minimum protein score of 22, minimum peptide score of 15, and maximum expectation values of 0.01 for protein and 0.001 for peptide matches, resulting in a protein false discovery rate of 0.4%. Protein identification results from specific TAP experiments were reported with a spectral count as an approximation of protein abundance, along with percent sequence coverage and an expectation value for the probability of protein identification [40], [41].

### RNA Interference

Target gene fragments were selected based on default settings of the RNAit software [42]. They were amplified from the strain 29-13 genomic DNA with PCR using sense primer carrying flanking BamHI/HindIII sites and antisense primer carrying flanking XhoI/XbaI sites for cloning the individual fragments into the Stem-loop pALC14 plasmid [24], [43]. The constructs were linearized with NotI and transfected into 29-13 procyclic cells via electroporation [34]. The transfected cells were cultivated for 15 to 18 days, and the stable transfectants were selected in the presence of 1 µg ml^−1^ puromycin. Clonal cell lines were established by limiting dilution and cultivation in 24-well plate. The cloned stable transfectants that reached a constant growth rate after at least three regular passages were selected and tested for RNAi phenotypes. To induce RNAi, 1 µg ml^−1^ tetracycline was added to the culture of a cell density of 2×10^5^ cells ml^−1^. Cell growth was monitored by counting the cell numbers with a haemocytometer at 24 hr intervals. For RNA extraction and cell cycle analysis, 24 hr cell samples after RNAi induction were collected, washed once with PBS and processed for either RNA extraction using the TRIzol® reagent (Invitrogen) or cell cycle analysis in flow cytometry (see below).

### Quantitative Real-time RT-PCR (qRT-PCR)

The first strand cDNA was generated from the RNA samples using iScript RT kits (Bio-Rad). qPCR was performed on the cDNA using gene-specific primer sets that are different from the primer sets for the RNAi DNA construct. It was performed using the SsoFast SYBR® Green supermix (Bio-Rad) with the quantitative analysis and statistical significance empirically calculated by the CFX Manager™ software (Bio-Rad). For loading controls, 100-bp products of Paraflagellar rod-A (PFR-A) and α-tubulin genes were amplified in the same qPCR assay.

### Flow Cytometry

Flow cytometry was performed on propidium iodide (PI)-stained cells to determine the DNA contents of the cells. At each 24 hr interval after RNAi-induction, cells were fixed, stained and processed for fluorescence activated cell sorting scan (FACScan) analysis according to the previously established protocol [24]. The DNA peaks of PI-stained cells were analyzed with the FACScan analytical flow cytometer (BD Biosciences). FL2-A DNA peaks were calculated using CellQuest software (BD Biosciences). Cells were also harvested, washed once with PBS, attached to Poly-L-Lysine coated cover slips and mounted in vectashield medium with DAPI (Vector Lab) for microscopic examination of nucleus (N)/kinetoplast (K) configurations. Percentages of cells with different N&K configurations in each sample were determined by counting at least 200 cells with an epifluorescence microscope.

### Epitope Tagging of Endogenous Proteins in Procyclic-form *T. brucei*

The 3′-terminal fragment of CDC27, AP1 and AP2 genes were amplified by PCR and cloned into the pC-PTP-NEO plasmid [35]. The three resulting DNA constructs, pC.CDC27.PTP, pC.AP1.PTP and pC.AP2.PTP were linearized with AvaI, BbsI and XhoI, respectively, and transfected into the strain 427 cells by electroporation. Stable clonal transfectants were selected under 40 µg ml^−1^ G418. For 3HA-epitope tagging of the endogenous mitotic cycB2/cyc6 gene, the C-terminal fragment of the gene (DB accession number: Tb11.01.8460) was amplified from the genomic DNA using gene specific primers and cloned into the pC.3HA.Bla plasmid, which is a modified version of the endogenous targeting plasmid pC.PTP.NEO [35], and was transfected into either 29-13 procyclic cells or 29-13 cells with a stably maintained AP2 RNAi plasmid. Transfectant selection was carried out under 10 ug ml^−1^ Blasticidin.

### Western Blotting

Cells were harvested, washed twice in phosphate-buffered saline (PBS), lysed by sonication in SDA-PAGE laemmli sample buffer and cleared by a brief centrifugation. Samples were fractionated on SDS-PAGE and transferred onto PolyVinylidene DiFluoride (PVDF) membrane (Bio-Rad, CA). After blocking in TBST (20 mM Tris-HCl [pH 7.4], 150 mM NaCl, 0.1% Tween 20) containing 5% skim milk, the immuno-blot membrane was probed with primary anti-Prot C HPC4 monoclonal antibodies (Roche diagnostics, CA) and stained with the secondary anti-mouse IgG-HRP conjugate (Sigma, MO).

### Immunoprecipitation

Cells were harvested, washed once in PBS and the cells extracted in the lysis buffer (25 mM Tris-Cl, pH 7.6, 100 mM NaCl, 1% Nonidet P-40, 1 mM dithiothreitol, and protease inhibitor cocktail) for 30 min on ice. After being cleared by a brief centrifugation, the lysate was incubated with anti-HA mAb (Sigma, MO) at 4°C for 60 min and then with IgG Sepharose beads overnight. The beads were sedimented by a brief centrifugation, washed in PBS, cooked in SDS-PAGE sample buffer and fractionated on SDS-PAGE. The gel was immuno-blotted onto PVDF membranes (Bio-Rad, CA), and the HA tag was probed with anti-HA HRP-conjugated mAb (Sigma, MO).

## Results

### Functional Divergence between Yeast and *T. brucei* APC/C Subunits

**Figure 1:**
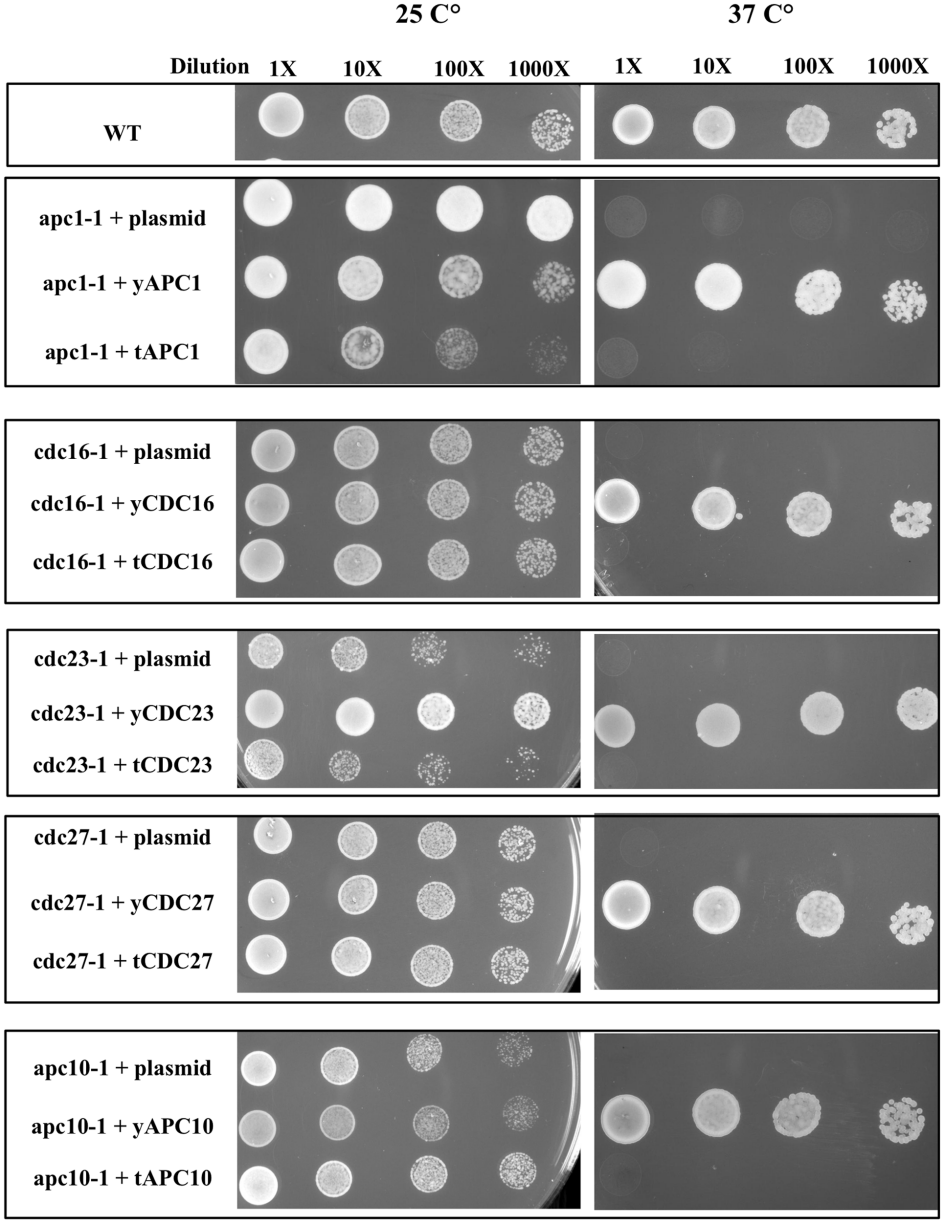
Functional divergence between *T. brucei* and yeast APC/C subunits. Yeast complementation assay. Temperature-sensitive (*ts*) yeast mutants of APC/C subunit genes *apc1-1*, *cdc16-1*, *cdc23-1*, *cdc27-1* and *apc10-1* were transformed with pRS416-ADH plasmids expressing corresponding yeast (y) or *T. brucei* (t) full-length APC/C genes. After transformation and selection, the cloned cells were grown either at the permissive (25°C) or the restrictive (37°C) temperature. Wild-type W303 (WT) cells and yeast *ts* mutant cells transfected with the empty vector (plasmid) were used as positive and negative controls, respectively.

It has been generally believed that a strong evolutionary conservation of APC/C function exists among APC/C subunit proteins in organisms as diverse as *Drosophila*, *Caenorhabditis elegans*, human and yeast [44], [45], [46]. Although sequence identities among the individual subunit genes are in the range of only 9.6 to 25.5% and sequence similarities in the range of only 21.5 to 54.5% (data not shown), Drosophila APC11 [45], C. elegans CDC26 [44] and human APC13 [46] were found capable of complementing their counterparts in yeast. T. brucei APC/C subunits have 13.4–23.0% sequence identity and 35.1–42.3% sequence similarity with the yeast counterparts, which are not significantly different from the other metazoa. In order to clarify if some of the T. brucei APC/C subunits are capable of replacing the essential functions of their yeast counterparts, we performed yeast complementation assays to cover a total of 5 S. cerevisiae temperature sensitive mutants *apc1-1*, *cdc27-1*, *cdc16-1*, *cdc23-1* and *apc10-1* [29] with the corresponding T. brucei subunit homologues. Results observed at the restrictive temperature showed that the growth of yeast mutant cells was fully rescued by homologous expression of the corresponding yeast genes (Figure 1). But heterologous expression of the corresponding T. brucei genes failed to rescue yeast cell growth in all 5 cases tested (Figure 1). Therefore, none of the five *T. brucei* APC/C subunit homologues was capable of complementing their counterparts in the yeast. There could be thus a significant structure-function discrepancy between the APC/C’s of the two organisms (see Discussion below).

### TAP of the APC1-PTP Protein Complex from *T. brucei*

In order to identify the intact APC/C core from T. brucei, the potential scaffold protein subunit APC1 in the complex [29], [47] was tagged with a PTP and expressed in the procyclic form of T. brucei via homologous genetic recombination. The APC1-PTP fusion protein was then gently isolated from the cell lysate using a well-established TAP procedure for purifying the entire APC/C from T. brucei [10], [35].

**Figure 2:**
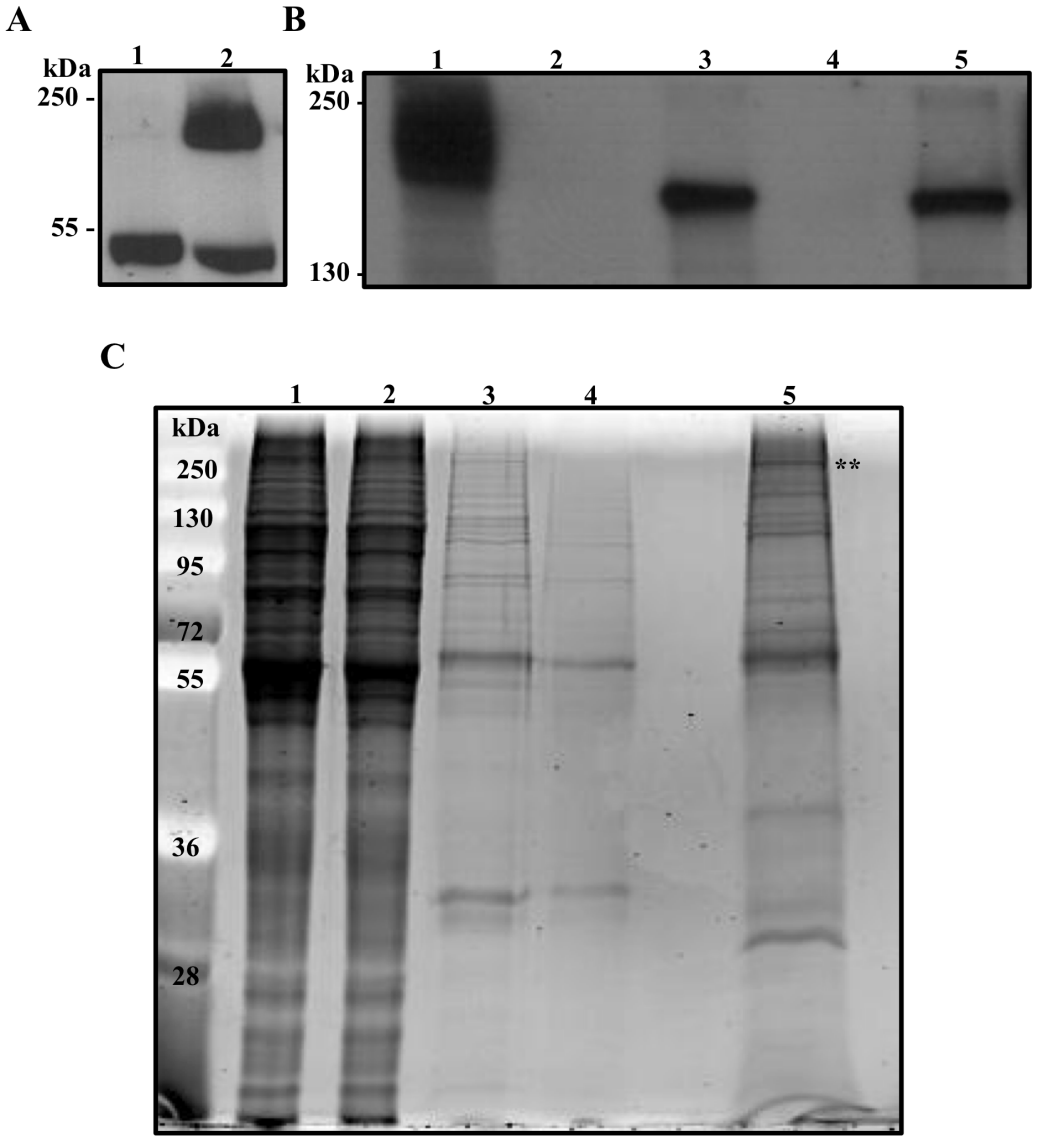
TAP of APC1-PTP and identification of APC1-associated proteins. (**A**) Lysates of strain 427 cells transfected either with empty vector (control, lane 1) or pC.APC1.PTP (lane 2) were fractionated on 10% SDS-PAGE gel, Western blotted and simultaneously probed with anti-protein C antibody (HPC4) and anti-tubulin. The blot was then detected with an anti-mouse HRP-conjugated secondary antibody. (**B**) Stepwise Western blot monitoring of APC1-PTP during TAP. Samples were analyzed from 1. IgG input (1x), 2. IgG-Sepharose flow-through (1x), 3. The elute from IgG-Sepharose after TEV-protease digestion (5x), 4. The flow-through from anti-protein C matrix (5x), and 5. The EGTA final elute (20x). The blot was probed with HPC4 antibody and the values (in x) represent the relative amounts of samples analyzed. (**C**) Samples collected from TAP as in (**B**) were fractioned on 10% SDS-PAGE gel and stained with SYPRO-Ruby. (**) on the top indicates the position of APC1-PTP fusion protein.

**Figure 3:**
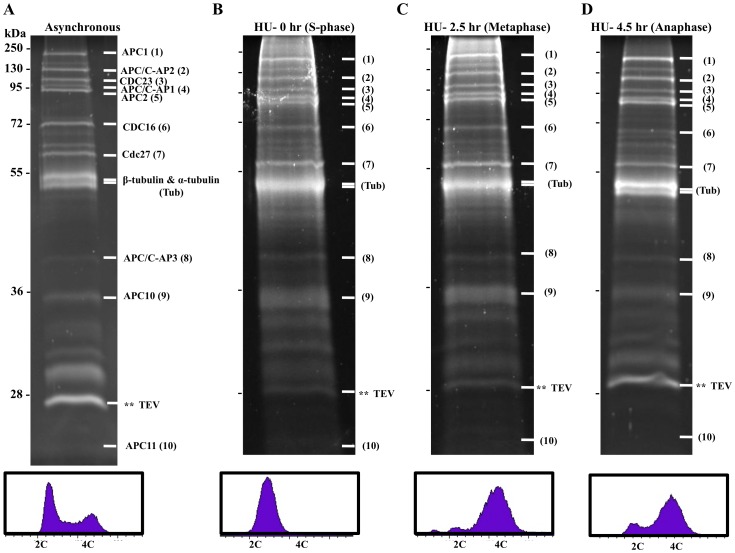
The *T. brucei* APC/C profiles during different phases of the cell cycle progression. The final EGTA elutes from TAP of APC1-PTP from (**A**) the G1 phase enriched cells; (**B**) The late S-phase cells; (**C**) The metaphase enriched cells and (**D**) The anaphase enriched cells were each fractioned in 10% SDS-PAGE and stained with SYPRO-Ruby. The individual protein bands identified by subsequent LC-MS/MS analysis are indicated on the right-hand side of each gel panel with the molecular masses (in kDa) indicated on the left-hand side. Tub and TEV denote contaminating tubulins and TEV protease, respectively. The cell samples were also analyzed by flow cytometry and the histograms of cellular DNA contents are displayed below the corresponding SDS-PAGE panels.

A stably transfected procyclic-form cell line of T. brucei strain 427 expressing APC1-PTP was isolated and the expression of APC1-PTP in the crude lysate was confirmed with Western blotting (Figures 2A lane 2 and 2B, lane 1). During the TAP, Western blots indicated that the fusion protein was successfully bound to the IgG beads (Figure 2B, lane 2) and eluted after TEV protease digestion in a somewhat reduced molecular mass as anticipated (Figure 2B, lane 3). The cleaved fusion protein was then adsorbed to a column of anti-protein C beads (Figure 2B, lane 4) and eluted effectively with EGTA (Figure 2B, lane 5) resulting in a final preparation of purified APC/C. Samples from each step of purification were examined with SDS-PAGE stained with SYPRO-Ruby (Figure 2C). The stained gels showed consistent changes of protein profiles through each step of purification with a significant purification of the protein sample achieved after TEV protease digestion and elution from IgG-beads (Figure 2C, lanes 2 & 3). Further enrichment and elimination of individual proteins were achieved after adsorption to the anti-protein C beads and elution with EGTA. The protein profile of the final product analyzed with SDS-PAGE (Figure 2C, lane 5) was highly reproducible among several preparations of cells (comparing Figure 2C, lane 5 and Figure 3A).

The protein profile represents the likely APC/C composition from an asynchronous cell population, which is a population enriched with G1 cells up to 80% shown in flow cytometry (Figure 3A). The cells were then synchronized with 0.3 mM hydroxyurea by an established procedure [37] resulting in 100% of the cells arrested in late S-phase (Figure 3B). They were then released from hydroxyurea and allowed to grow synchronously for 2.5 hrs when 65% of the population was found to have reached the metaphase and 15% in the anaphase in flow cytometry and immunofluorescence assays using chromosome passenger complex 1 (CPC1) protein as the marker (Figure 3C, Figure S1) [10]. When the time period of synchronous growth was extended to 4.5 hrs, 65–70% of the cells were in the anaphase, whereas the rest of the cells had apparently gone beyond cytokinesis and reached the G1 phase (Figure 3D, Figure S1). The method thus enabled us to prepare large batches of procyclic-form T. brucei cells enriched in G1-phase, S-phase, metaphase and anaphase, respectively, and subject them to the TAP (Figure 3). The results from SDS-PAGE of the purified APC1-PTP protein complexes from the cells enriched in different phases of the cell cycle are presented in Figure 3 for direct comparisons. The protein profiles of each sample appear highly similar to one another, and the intensities of individual protein bands stained with SYPRO-Ruby in the gels appear also relatively unchanged among the four cell samples (Figure 3). This preliminary observation provides an indication that T. brucei APC/C remains relatively unchanged throughout the cell cycle without any association or dissociation with other protein(s). This tentative conclusion requires further verification with LC/MS/MS, which is capable of also identifying the individual APC1-PTP-assocaited proteins and compare them with the 7 APC/C subunit homologues previously found in T. brucei genome database [23].

### LC-MS/MS Analyses of *T. brucei* APC/C Derived from Different Phases of Cell Cycle

Samples of the APC1-PTP fusion protein complex purified from duplicate or triplicate cell samples, each enriched in different phases of the cell cycle, were fractionated in SDS-PAGE (Figure 3). Individual protein bands in the gels were sliced out, diced and digested with trypsin by the well-established procedures. As a control, lysate of 427 cells transfected with empty pC.PTP plasmid was purified by the same procedure and individual protein bands were sliced from the SDS-PAGE and processed for trypsin digest as mentioned above. Each digest was then fractionated with LC, and subjected to MS/MS analysis for peptide identification. The results in protein identities versus the individual protein bands in a SDS-PAGE gel, are represented in Figure 3A of a sample purified from a cell population enriched in G1 phase. Thirteen proteins are identified in the purified APC-PTP complex. Other than the persistent presence of α-tubulin, β-tubulin and TEV protease, which are also present in the control sample and are thus likely common contaminants (data not shown), the rest of the 10 proteins in the purified complex are found to include the 7 APC/C subunit proteins originally derived from T. brucei genome database plus 3 un-identified proteins designated the APC/C-Associated Proteins (AP); AP1, AP2 and AP3, respectively. The rest of the undefined protein bands have also been analyzed, and they were primarily degradation products from the 10 identified proteins and the high abundance background proteins.

These 10 identified proteins all appeared in the purified APC/C samples from the G1-phase (Figure 3A), S-phase (Figure 3B), metaphase (Figure 3C) and anaphase enriched cells (Figure 3D) at relatively unchanged quantities. There has been no other protein detected in any of the 4 purified APC/C samples, which suggests that there is no additional protein above the limit of detection that is associated with APC/C that could sustain the mild TAP experimental conditions during any phase of the cell cycle progression of T. brucei. The 10 proteins may thus constitute the core subunits of the APC/C in T. brucei.

More detailed data from the MS/MS analysis are presented in Table 1 and Table S1. The estimated molecular masses of the 7 APC/C subunits all agree well with those derived from the genomic database. All the proteins are each identified with adequate numbers of unique peptides and sufficient percentages of coverage in the two tables with but one exception with APC11; data on this protein are apparently missing in the samples from G1-phase and anaphase enriched cells. This is most likely attributed to the relatively low molecular weight of APC11 (10 kD) that facilitates diffusion of the protein from the gel during SDS-PAGE [48], [49]. This small protein is thus still considered an integral component of T. brucei APC/C.

**Table 1:** LC-MS/MS analysis of the APC/C samples purified from different phases of *T. brucei* cell cycle progression.

Accession number	Proteins	Molecular mass	G1-phase N = 3		S-phase N = 1		Metaphase N = 1		Anaphase N = 2	
(Tritryp_DB)	Names	kDa	# Unique peptides	% Coverage	# Unique peptides	% Coverage	# Unique peptides	% Coverage	# Unique peptides	% Coverage
Tbg972.6.3070	APC1	202.07	40	31	54	40	60	34	54	40
Tbg972.9.6840	APC2	85.89	31	49	34	58	47	62	34	58
Tbg972.6.1890	CDC16	67.26	29	50	26	55	42	57	26	55
Tbg972.1.2470	CDC23	99.69	31	42	24	42	42	48	24	42
Tbg972.10.12580	CDC27	62.87	11	24	9	19	15	26	9	19
Tbg972.10.19130	APC10	25.63	5	29	3	22	7	29	3	22
Tbg972.9.5890	APC11	9.79	0	0	2	28	0	0	2	28
Tbg972.11.6300	AP1	82.59	30	46	26	46	42	22	26	46
Tbg972.7.6680	AP2	116.48	25	27	31	43	32	34	31	43
Tbg972.8.3830	AP3	47.00	2	6	3	10	5	17	3	10

The number of unique peptides and percent sequence coverage are summarized across the replicate analysis as indicated. For a complete analytical report, please refer to Table S1.

To further verify that there is no significant fluctuation of the intracellular levels of APC/C subunits during cell cycle progression, APC1, CDC27, AP1 and AP2 were each tagged with PTP at the C-termini, integrated into chromosomes via homologous recombination and expressed in transfected cells as previously described. The transfected cells were then synchronized with hydroxyurea into late S-phase [37] and released for synchronized growth for 8 hrs, which covers the entire cell cycle of *T. brucei* [3]. Hourly samples were taken during the incubation and examined on Western blots. The results, presented in Figure S2, indicated little changes of the protein levels in the four samples, suggesting limited fluctuation in the level of APC/C subunits during cell cycle progression of procyclic-form T. brucei.

### Tentative Identification of AP1 as APC4

In NCBI BLAST analysis, the sequence of AP1 was successfully aligned with the protein fragments from the core APC/C subunit APC4 from several eukaryotic organisms including *Schizosaccharomyces pombe* and *Arabidopsis thaliana* (data not shown). By searching the domain database of Pfam for specific motifs, the most significant structural similarity between AP1 and the APC4’s was identified in the Apc4-WD40-like domain. The Pfam-A (CL0186) domain database identified a bit score of 11.2 and an E value of 0.18 for AP1. The WD40 domain in APC4 is an N-terminal propeller-shaped domain that serves to stabilize the interaction with APC5 in the APC/C complex [50]. All known APC4 proteins have been found to contain a single N-terminal WD40-like domain except for the *A. thaliana* and *T. brucei* homologues, each of which contains two WD-40-like domains (data not shown). This finding was further confirmed by a reciprocal BLAST analysis searching the TriTryp database with yeast and vertebrate APC4 sequences. AP1 is thus re-designated APC4.

AP2 and AP3 remain unidentified after extensive BLAST and domain search analysis. But their orthologues have been found in the genomic databases of *Trypanosoma cruzi* and *Leishmania major* (data not shown), suggesting that they could be the common core subunits of APC/C among all Kinetoplastidae.

### RNAi Knockdown of AP2 Arrested the Cells in G2/M Phase

**Figure 4:**
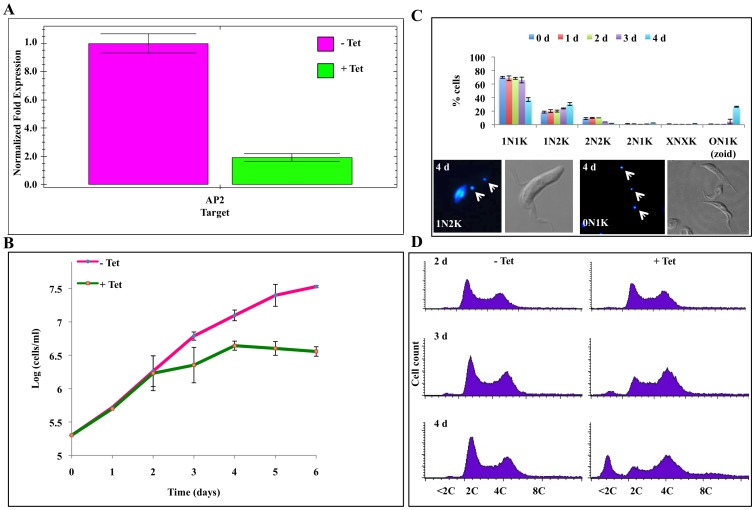
RNAi knockdown of AP2 in *T. brucei* procyclic cells. (**A**) Specific AP2 mRNA depletion was assayed with qPCR after RNAi induction for 72 hours. Data are derived from two independent experiments with standard deviation (±SD) error bars displayed. (**B**) The rate of cell growth after RNAi induction. The mean ±SD values are derived from two independent experiments. (**C**) Time samples (d, days) of the RNAi-induced cells were stained with DAPI and analyzed with a microscope for numbers and configurations of nucleus (N)/kinetoplast (K) among individual cells. Data are presented as percentages of cells from a total number of ∼200 cells at each time point from two independent induction experiments. Error bars represent the standard deviation (SD) are presented. Representative DAPI-stained 1N2K and 0N1K cells after 4 days of RNAi induction are shown (lower panel). (**D**) Time-dependent (d, days) changes of DNA profiles in the RNAi-induced cells were analyzed by flow cytometry.

Expression of the three newly identified APC/C subunits was each knocked down with RNAi for potential cell phenotype. By inducing target-specific RNAi in procyclic-form cells [33], specific mRNAs were significantly depleted. Data from qPCR showed that APC4 mRNA was almost totally depleted (Figure S3A), AP2 transcript was reduced more than 80% (Figure 4A), whereas AP3 mRNA was also knocked down by 80% (Figure S4A). Only the knockdown of AP2, however, turned out to demonstrate a clear phenotype; the cells continued to grow for two more days following the RNAi induction, but apparently ceased growing thereafter (Figure 4B). Microscopic examination of DAPI-stained cells showed a gradual increase of cells containing 1 nucleus and 2 kinetoplasts (1N2K) and a slow reduction in the number of 2N2K cells when compared with the control (Figure 4C). The elongated nucleus and two well segregated kinetoplasts in the 1N2K cells suggest that the cells are arrested in metaphase [3], [51], [52] (Figure 4C, left lower panel). There was also a dramatic increase of 0N1K cells (zoids) (Figure 4C, right lower panel) up to 25-30% of the total cell population on day 4 of the RNAi induction. This is a hallmark of the procyclic cells arrested during mitosis, in which cytokinesis and cell division still carries on, generating anucleated cells [4], [6]. All these data thus provide a strong indication that the procyclic-form cells are arrested during mitosis, likely in the metaphase, following a knockdown of AP2. Flow cytometry indicated a diminished 2C cell numbers and an increase of 4C cell population plus a prominent band of cells with less than a 2C DNA content after 4 days of RNAi, most likely the zoids (Figure 4D). Thus all the experimental data point to a blocked mitosis, which are highly similar to the data from a knocking down of APC1 or CDC27 in our previous study, showing that the procyclic-form T. brucei was arrested in the metaphase [23]. Being a likely subunit of APC/C, the knockdown of AP2 may also result in a blocking of metaphase-anaphase transition.

APC4 and AP3 were also knocked down, but the results, presented in Figures S3 and S4, indicated no clear phenotype. They are thus classified as the APC/C subunits like APC2, CDC16, CDC23, APC10 and APC11; whose knockdowns from T. brucei did not register a phenotype [23].

### AP2 Depletion Stabilized the Mitotic Cyclin CycB2/cyc6

Following our tentative identification of the composition of APC/C in T. brucei, the next question concerned the potential function of this protein complex. Since a homologue of securin/Pds1 has not yet been found in T. brucei, we focused our attention on another commonly known substrate of APC/C; the mitotic cyclin B, whose destruction by the combined actions of APC/C and 26S proteasome toward the late phase of mitosis is required for mitotic exit in yeast and metazoa [22], [53]. A functional homologue of mitotic cyclin B, cycB2/cyc6, has been identified in T. brucei [4], [28]. It is involved in activating the mitotic CDK cdc2-related-kinase 3 (CRK3) during mitosis [4]. An RNAi knockdown of cycB2/cyc6 arrested T. brucei in the G2/M phase [28], though a potential disappearance of cycB2/cyc6 from T. brucei in the late phase of mitosis has not yet been studied.

**Figure 5:**
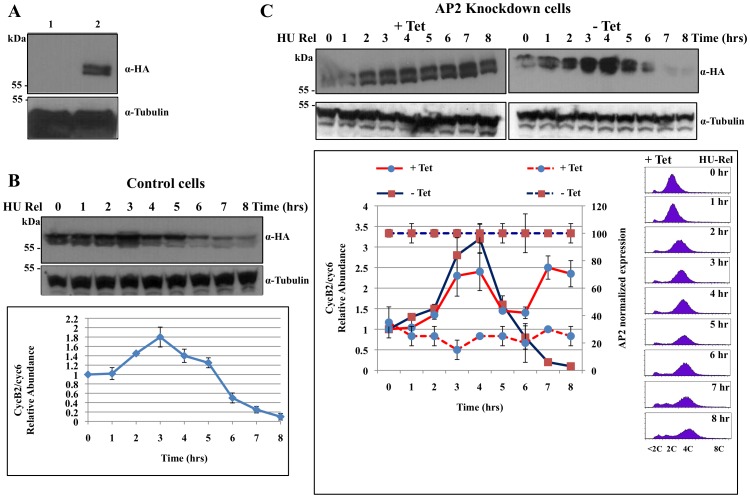
Effect of AP2 knockdown on the expression of CycB2/cyc6 in *T. brucei.* (**A**) Western blot analysis of the cell lysates of; 1. wild-type cells; and 2. the cells expressing CycB2/cyc6-3HA. The blot is stained with anti-HA mAb and the anti-tubulin antibody was used as a loading control. (**B**) The Control Cells; Cells expressing CycB2/cyc6-3HA were synchronized in late S-phase with hydroxyurea and released for synchronous growth for 8 hrs to complete a cell cycle. Hourly cell samples were lysed and fractionated in SDS-PAGE, immuno-blotted and probed with anti-HA mAB for CycB2/cyc6-3HA expression. The time course of level changes of CycB2/cyc6-3HA was quantified against tubulin control using the ImageJ software and presented in the lower panel. The relative abundance of CycB2/cyc6 has a value set at 1 from the zero time point. Error bars represent the SD from two independent experiments. (**C**) AP2 RNAi cells expressing CycB2/cyc6-3HA were induced (+Tet) for RNAi for 48 hours, and then synchronized to late S-phase with hydroxyurea and released for synchronous growth for 8 hours while the RNAi was maintained. CycB2/cyc6-3HA expression was monitored as described in (B). RT-PCR analysis of AP2 transcript levels in the hourly cell samples was performed. They and the levels of CycB2/cyc6 from the Western analysis are plotted versus time and presented in the lower left panel. Error bars represent the SD from two independent experiments. The same time samples from AP2 RNAi induced culture (+ Tet) after hydroxyurea release were analyzed by flow cytometry and the data are presented in the lower right panel.

Since an APC2 knockdown in mice and an APC16 depletion in human cells were reported to stabilize the mitotic cyclin B [48], [54], we knocked down the expression of AP2, and monitored the fate of CycB2/cyc6 thereafter. To do this, one of the two endogenous alleles encoding CycB2/cyc6 was tagged with 3HA through homologous recombination and the expression of CycB2/cyc6-3HA was verified on Western blot (Figure 5A).

The cells expressing CycB2/cyc6-3HA were treated with 0.3 mM hydroxyurea for 16 hrs for synchronization of the cells in late S-phase, and were released for synchronous growth. Hourly cell samples were taken for Western blot analysis. The results (Figure 5B) showed that following an initial increase in the beginning 3 hrs, when the cells entered from the late S-phase into the metaphase (see Figure 3 and Figures S1 & S5), there was a steady decrease in the level of CycB2/cyc6 thereafter. The decrease continued up to 8 hrs when a full cell cycle was completed [3]. CycB2/cyc6 is thus confirmed to disappear like the other mitotic cyclins in the late phase of mitosis.

To verify if the APC/C in T brucei is involved in regulating the turnover of CycB2/cyc6, the same experiment was repeated using the AP2 RNAi cell line. In the control cells without RNAi induction, the time course of CycB2/cyc6-3HA level change in the synchronized cells paralleled that observed in Figure 5B except that the heightened level of the cyclin was extended a little longer to 4 hours before the decline (Figure 5C), which could be attributed to slightly different physiological conditions between different batches of synchronized cells. When AP2 RNAi was induced for 48 hrs (see Figure 4B) and the cells were then subjected to hydroxyurea synchronization and released for synchronous growth, expression of AP2 was knocked down as anticipated throughout the synchronization experiment (Figure 5C). The level of CycB2/cyc6 reached the high plateau within 3 to 4 hrs, reduced by about 30% after 5 to 6 hrs, but restored to the original high level after 7 to 8 hours (Figure 5C). Comparing with the no RNAi induction control, it is clear that a knockdown of AP2 expression resulted in stabilization of CycB2/cyc6 in the late mitotic phase of T. brucei. The progression of synchronized AP2 knockdown cells was also analyzed with flow cytometry and the data are presented in the lower right panel of Figure 5C. When these data are compared with those from the progression of synchronized wild-type cells in Figure S5, it is clear that the progression of AP2 knockdown cells is arrested in the G2/M phase. Since AP2 is a core subunit of APC/C, it is most likely that the function of APC/C is required for the destruction of CycB2/cyc6 and passage of cells beyond mitosis. From the knowledge derived from other eukaryotes, it is reasonable to postulate that the APC/C of T. brucei functions as an E3 ligase that poly-ubiquitinates CycB2/cyc6 and subjects it to 26S proteasome degradation.

### The Mitotic Cyclin in *T. brucei* is Likely Poly-ubiquitinated by APC/C and Degraded by 26S Proteasome during Mitosis

**Figure 6:**
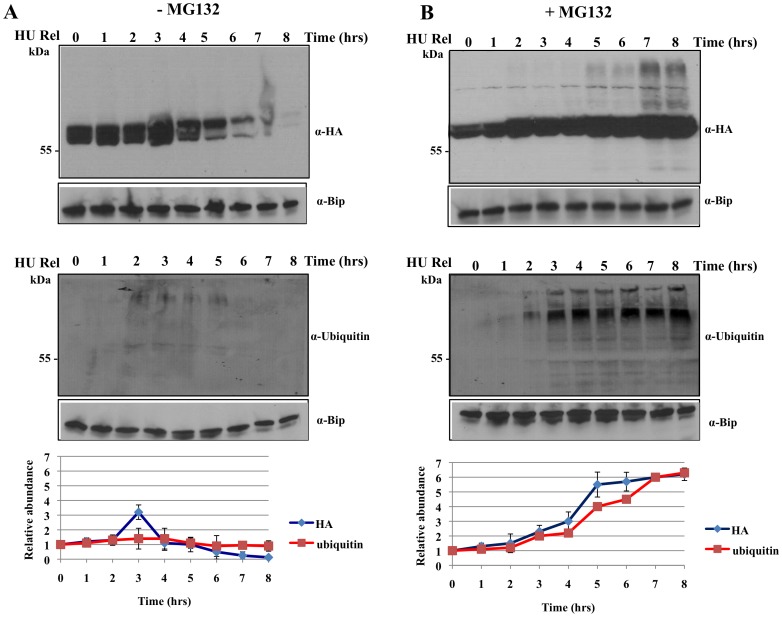
Polyubiquitination and proteasome degradation are involved in the turnover of CycB2/cyc6 during mitosis. Cells arrested in late S-phase by hydroxyurea treatment were released for synchronous growth for 8 hours in the absence (**A**) or the presence (**B**) of 20 µM MG132. Hourly cell samples were lysed, subjected to immunoprecipitation with anti-HA mAb and fractionated with SDS-PAGE. Western blotting was used to analyze the time course of the level of CycB2/cyc6-3HA with anti-HA mAb (upper panels) or polyubiquitination staining with anti-Ubiquitin (Sigma, U5379) antibody (middle panels). Anti-Bip was used as loading controls. A quantitative analysis of CycB2/cyc6-3HA relative abundance and ubiquitin staining intensity for each experiment were plotted versus time in the bottom panels. Error bars represent the SD from two independent experiments.

The presence of 26S proteasome and the structure and function of this protein complex in T. brucei have been thoroughly investigated and identified by us in our previous studies [34], [55]. In order to verify if the turnover of CycB2/cyc6 depends on the function of proteasome in T. brucei, the latter was treated with a reversible inhibitor of proteasome, MG132, at 20 µM known to totally inhibit the proteasome activity in T. brucei [56]. Cells expressing CycB2/cyc6-3HA and synchronized with hydroxyurea were released for synchronous cell cycle progression in the presence of MG132. Hourly cell samples were taken for immunoprecipitation with anti-HA followed by analysis on Western blot stained with the antibodies to HA or ubiquitin. The results showed that while there is a drop of CycB2/cyc6 level in the cells after 3 hrs without MG132 treatment as anticipated (Figure 6A, upper panel), it keeps increasing up to 6-fold of the original value after 8 hrs of growth in the presence of MG132 (Figure 6B, upper panel). Apparently, the proteasome function is required for the degradation of CycB2/cyc6 during mitosis of *T. brucei*. The time-dependent accumulation of ladders of higher molecular mass bands in the MG132 treated samples suggests also formation of poly-ubiquitinated CycB2/cyc6 (Figure 6B, upper panel).

When the Western blot of the anti-HA immunoprecipitate was stained with anti-ubiquitin, there was little specific staining for poly-ubiquitinated proteins while MG132 was not present (Figure 6A, middle panel). In the presence of MG132, however, there was a steady increase in the intensity of a ladder of protein bands on top of the CycB2/cyc6 band beyond the mitotic phase of the cells (Figure 6B, middle panel). They are most likely the poly-ubiquitinated CycB2/cyc6 that cannot be degraded when the proteasome activity is inhibited. It is probable that the poly-ubiquitinated CycB2/cyc6 is a product of the APC/C action.

## Discussion

In the present study, we indicated that among the five APC/C subunit homologues in T. brucei; APC1, CDC16, CDC23, CDC27 and APC10, none was capable of complementing the function of their counterparts in yeast. Although APC/C has a conserved presence and function among all the eukaryotes examined thus far, the sequences of individual APC/C subunit proteins do not appear to be highly conserved [12]. Drosophila APC11 [45], C. elegans CDC26 [44] and human APC13 [46] have been, however, tested in the yeast complementation assays and found capable of substituting the corresponding subunits in yeast. It makes thus the negative outcome from testing all 5 T. brucei subunits a little difficult to explain from a simple view on protein sequence discrepancies. An alternative explanation could be by postulating a distinctive mechanism of inter-subunit interactions in constituting the APC/C in T. brucei, i.e., T. brucei APC/C subunits may be incapable of incorporating into yeast APC/C.

The assembly and three-dimensional structure of APC/C remain poorly understood for the time being [57]. Three-dimensional electron microscopic structural analysis of yeast APC/C located APC1 in an L-shaped rod that links APC2 to CDC23, whereas CDC23 is connected to APC5 with APC4 interconnecting APC1 and APC5 [58], [59]. APC1, the largest APC/C subunit, consists of 11 highly repetitive 35–40 amino acid proteasome-cyclosome (PC) sequences at the C-terminus. This PC motif is shared with the RPN1 and RPN2 subunits of the proteasome 19S regulatory particle [60], and is assumed to be the main binding sites for other subunits in forming APC/C. APC/C purified from the yeast mutant *apc1Δ* was found lacking association among the majority of other subunits [29]. APC1 is thus classified as the major scaffold protein in yeast APC/C [29]. These 11 PC repeats are, however, absent from T. brucei APC1 (data not shown), which could provide a supporting evidence that T. brucei APC/C may have a mechanism of assembly highly distinctive from that in yeast. This distinction may explain why the APC/C subunits from T. brucei are not complementary to the corresponding subunits missing from the yeast.

The outcome from our TAP and LC-MS/MS analysis of APC1-PTP protein complex indicates that T. brucei APC/C is made of 10 core subunit proteins. The composition and level of APC/C remain apparently unchanged throughout the entire cell cycle of procyclic-form T. brucei. An even more intriguing finding was that neither CDC20 nor MCC complex proteins were found associated with APC/C during any phase of the cell cycle. This is in contrast to that observed among the other eukaryotes. An intact MCC complex with CDC20 protein had been co-purified with APC/C subunits using a similar TAP procedure in human cells [48]. Also, CDC20 was detected in the mitotic-enriched APC/C-TAP sample from the budding yeast and fission yeast [47]. In budding yeast, the MCC components MAD1, MAD2 and MAD3 were co-immunoprecipitated with epitope-tagged CDC20 throughout different stages of the cell cycle [61]. A common factor enabling all the complex formations mentioned above is CDC20. The fact that the T. brucei CDC20 homologue is not associated with APC/C at all during all phases of the cell cycle shows that it is not performing the function of mediating a binding of APC/C to MCC or activating APC/C to poly-ubiquitinate mitotic cyclin CycB2/cyc6 [62]. Our previous finding that an RNAi knockdown of CDC20 showed no detectable cell phenotype (data unpublished) tends to support this conclusion. The CDC20 homologue in T. brucei could be a structural homologue but not a functional one.

Our findings that there is no apparent structural homologues of MCC subunits in T. brucei genomic database (data not shown) and that no detectable protein was found associated with APC/C in the metaphase and dissociated from it in the anaphase of T. brucei provide a strong indication that this organism may not have a similar mechanism of regulating metaphase-anaphase transition as observed in other eukaryotes [63], [64]. A similar observation has been also made in the budding yeast, in which neither the MCC subunit protein MAD2 nor the spindle assembly checkpoint complex is required for normal cell growth [65], [66]. This peculiarity was attributed to the persistent presence of mitotic spindles throughout the yeast cell cycle [65]. It may not require specific spindle assembly prior to mitosis to facilitate capture of the mitotic microtubules by the kinetochores in chromosomes and bi-orientations of the chromosomes on the mitotic spindle [65]. T. brucei is an even more primitive organism than yeast and may not maintain an active regulation of spindle assembly either. This postulation may explain the apparent absence of a MCC-like complex in T. brucei. But the mechanism of activation of APC/C in triggering the metaphase to anaphase transition remains still unclear. The question whether a functional homologue of securin/Pds1 is present in T. brucei requires still an answer.

Other than the 7 subunits already identified in T. brucei APC/C genome DB [23], three additional subunit proteins, APC4, AP2 and AP3, were identified in this protein complex. AP2 and AP3 cannot find homologous proteins in all the genomic databases other than those of Kinetoplastidae. Searches for common motifs in APC/C subunits such as PC repeats, cullin homology, TPR, RING H2 and WD40/IR in these two proteins also turned out negative results. AP2 and AP3 could thus be specific subunits of only the APC/C’s among the Kinetoplastidae.

Among the 10 core subunits identified in T. brucei APC/C, only a knockdown of three of them, APC1 [23], CDC27 [23] and AP2, each resulted in an arrest of T. brucei procyclic-form cells in the metaphase. In budding yeast, 8 of the 13 APC/C subunits (APC1, APC2, APC4, APC5, APC11, CDC16, CDC23, CDC27) were found indispensible for viability. Their depletion abrogates the APC/C catalytic activity and blocks yeast cell cycle progression [25], [50], [67], [68]. The rest of the subunits are either non-essential (APC9, CDC26, SWM1, MND2) [25], [46], or their loss (APC10/DOC1) affects only the complex integrity or the rate of substrate binding and processing [69], [70]. SWM1 and CDC26 only have essential roles at restrictive temperatures in maintaining structural stability of the complex [26], whereas SWM1 and MND2 are essential during meiotic cell division [27]. In *S. pombe*, individual knockdowns of APC14 and APC15 did not display any abnormal phenotype but the cells developed a temperature–sensitive phenotype and chromosome segregation abnormalities when two proteins were mutated simultaneously or depleted with other APC components [47]. The fact that 7 out of 10 *T. brucei* APC/C subunits are dispensable for cell cycle progression could mean that they have redundant structural and functional roles with the other subunits. It may require double or multiple knockdowns of these subunits to inflict a detectable phenotype.

Despite all the apparent structural and functional uniqueness, *T. brucei* APC/C showed also some conserved functions as those observed in the other eukaryotes. The knockdowns of APC1, CDC27 and AP2 were each found to arrest the procyclic form T. brucei cells in metaphase, suggesting that the APC/C function is required for metaphase-anaphase transition. The APC/C function is also needed for degradation of the mitotic cyclin CycB2/cyc6 during mitosis in T. brucei. The degradation, essential for mitotic exit among the other eukaryotes [16], [22], is mediated by the 26S proteasome in *T. brucei*, which recognizes poly-ubiquitinated CycB2/cyc6 as substrate. This ubiquitination-dependent degradation is likely provided by the poly-ubiquitinating action of APC/C on CycB2/cyc6, a function that apparently remains conserved in T. brucei.

We have thus characterized an APC/C in T. brucei that performs apparently both of the well-known functions during mitosis. But its unusually simple composition and the apparent functional redundancy among the 10 subunits distinguish it from the other APC/C’s. The lack of an MCC mediated regulatory mechanism and the apparent absence of securin/Pds1 and CDC20 in T. brucei further demonstrates a significant discrepancy between T. brucei APC/C and the others. The APC/C in *T. brucei* could be thus easily classified as a potential target for anti-trypanosomiasis chemotherapy.

## Supporting Information

Figure S1 **Hydroxyurea (HU) synchronization of the cell cycle progression in** ***T. brucei*** **.** (**A**) Western blotting of TbCPC1-eYFP expression in TbAPC1-PTP cells. The same cell extract was immuno-probed with HPC4 antibody for PTP expression and anti-GFP antibody for eYFP expression. Asterisk indicates a non-specific anti-GFP immune-reactive band and upper and lower arrows indicate the positions of APC1 and CPC fusion proteins, respectively. (**B**) Flow cytometric analysis of hydroxyurea synchronized cells at 0, 2.5 and 4.5 hours after release. (**C**) Fluorescence microscopic analysis of cells co-expressing APC1-PTP and CPC1.eYFP at S-phase (0 hr), metaphase (2.5 hr) and anaphase (4.5 hr) after hydroxyurea release. DIC, DAPI and YFP filters are shown with merge composite. Bars = 2 µM. (**D**) Quantitative microscopic analysis of eYFP signals. Approximately 200 cells from each sample were counted and data are presented as localization pattern of S-phase (0 hr), metaphase (2.5 hr) and anaphase (4.5 hr) from two independent experiments.
(TIF)

Figure S2 **Time courses of expression of APC1, CDC27, AP1 and AP2 in synchronized** ***T. brucei*** **growth.** Cells expressing endogenous PTP fusion proteins of (**A**) APC1; (**B**) CDC27; (**C**) AP1 and (**D**) AP2 were arrested in late S-phase after 16 hr treatment with 0.3 mM hydroxyurea. Samples of the released cells were taken every hour and their lysates monitored for the expression of individual fusion proteins by immunoblotting using the HPC4 antibody with anti-tubulin antibody used as loading controls.
(TIF)

Figure S3 **The RNAi knockdown of AP1/APC4.** (**A**) qPCR assay of the level of AP1/APC4 mRNA 72 hrs after the induction of AP1/APC4 RNAi. (**B**) The rate of cell growth was monitored for 7 days after the RNAi induction. (**C**) N/K tabulations of the AP1/APC4-depleted cells on days 0, 1, 3 and 5 after RNAi induction. (**D**) Flow cytometric analysis of DNA contents in AP1/APC4-depleted cells. Little distinction was observed in the results from RNAi-induced and un-induced cells.
(TIF)

Figure S4 **RNAi knockdown of AP3. Panels A, B, C and D are as described in Figure S3.**
(TIF)

Figure S5 **Synchronization of the cell cycle progression in** ***T. brucei*** **with hydroxyurea.** Strain 29-13 procyclic *T. brucei* cells expressing CycB2/cyc6-3HA were treated with 0.3 mM hydroxyurea for 16 hours, washed twice in fresh medium and allowed to progress synchronously for 8 hours. The hourly cell samples were stained with propidium iodide, processed for flow cytometry and the FL2-A DNA peaks are presented. DNA contents (2C or 4C) were shown at the bottom.
(TIF)

Table S1 **Identification by LC-MS/MS of APC/C subunit proteins.** Protein components of the APC/C complex were identified by comparison of replicate LC-MS/MS peptide sequencing analyses. To be considered a candidate APC/C subunit, it was required that the protein was observed in all four cell cycle stages (G1, S-phase, Metaphase and Anaphase), that at least one experiment included a minimum of two unique peptides for confident protein identification, and that the protein was not observed in a control experiment.
(XLS)
