# Correction: A Minimal Anaphase Promoting Complex/Cyclosome (APC/C) in *Trypanosoma brucei*

**DOI:** 10.1371/annotation/d048ae1b-6a9b-4353-bdf2-c707925ce37c

**Published:** 2013-10-28

**Authors:** Mohamed Bessat, Giselle Knudsen, Alma L. Burlingame, Ching C. Wang

An affiliation for the first author was incorrectly omitted from the byline. In addition to institution number 1, Mohamed Bessat is also affiliated with the following: Department of Parasitology, Faculty of Veterinary Medicine, Alexandria University, Alexandria, Egypt. 

