# Supplementary figures and images for: A Minimal Anaphase Promoting Complex/Cyclosome (APC/C) in Trypanosoma brucei
Source: PLoS One. 2013 Mar 22;8(3):e59258. doi: 10.1371/journal.pone.0059258 (PMC3606461; doi:10.1371/journal.pone.0059258)

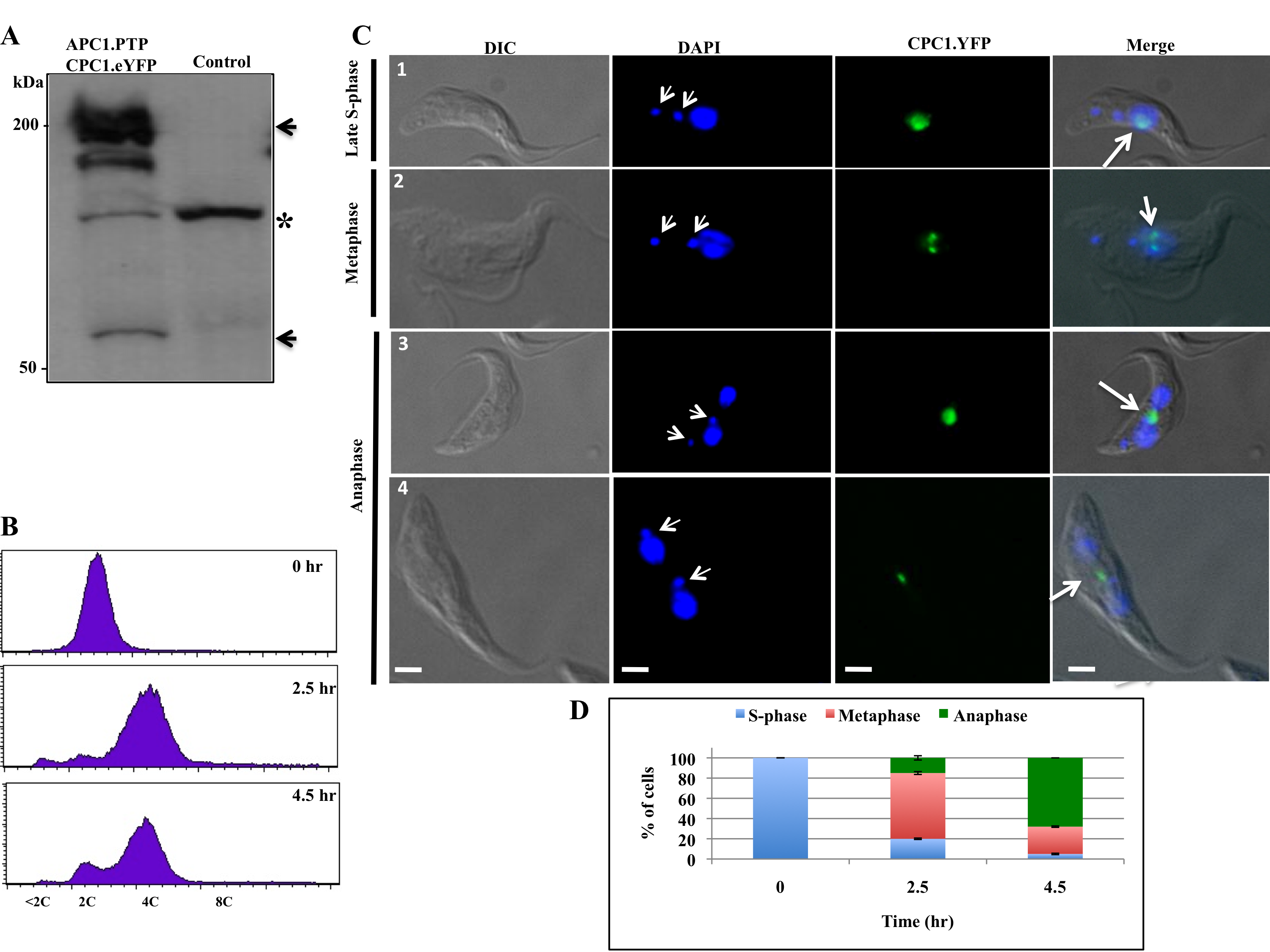

Supplement: Figure S1 — Hydroxyurea (HU) synchronization of the cell cycle progression in T. brucei . (A) Western blotting of TbCPC1-eYFP expression in TbAPC1-PTP cells. The same cell extract was immuno-probed with HPC4 antibody for PTP expression and anti-GFP antibody for eYFP expression. Asterisk indicates a non-specific anti-GFP immune-reactive band and upper and lower arrows indicate the positions of APC1 and CPC fusion proteins, respectively. (B) Flow cytometric analysis of hydroxyurea synchronized cells at 0, 2.5 and 4.5 hours after release. (C) Fluorescence microscopic analysis of cells co-expressing APC1-PTP and CPC1.eYFP at S-phase (0 hr), metaphase (2.5 hr) and anaphase (4.5 hr) after hydroxyurea release. DIC, DAPI and YFP filters are shown with merge composite. Bars = 2 µM. (D) Quantitative microscopic analysis of eYFP signals. Approximately 200 cells from each sample were counted and data are presented as localization pattern of S-phase (0 hr), metaphase (2.5 hr) and anaphase (4.5 hr) from two independent experiments. (TIF) [file pone.0059258.s001.tif]

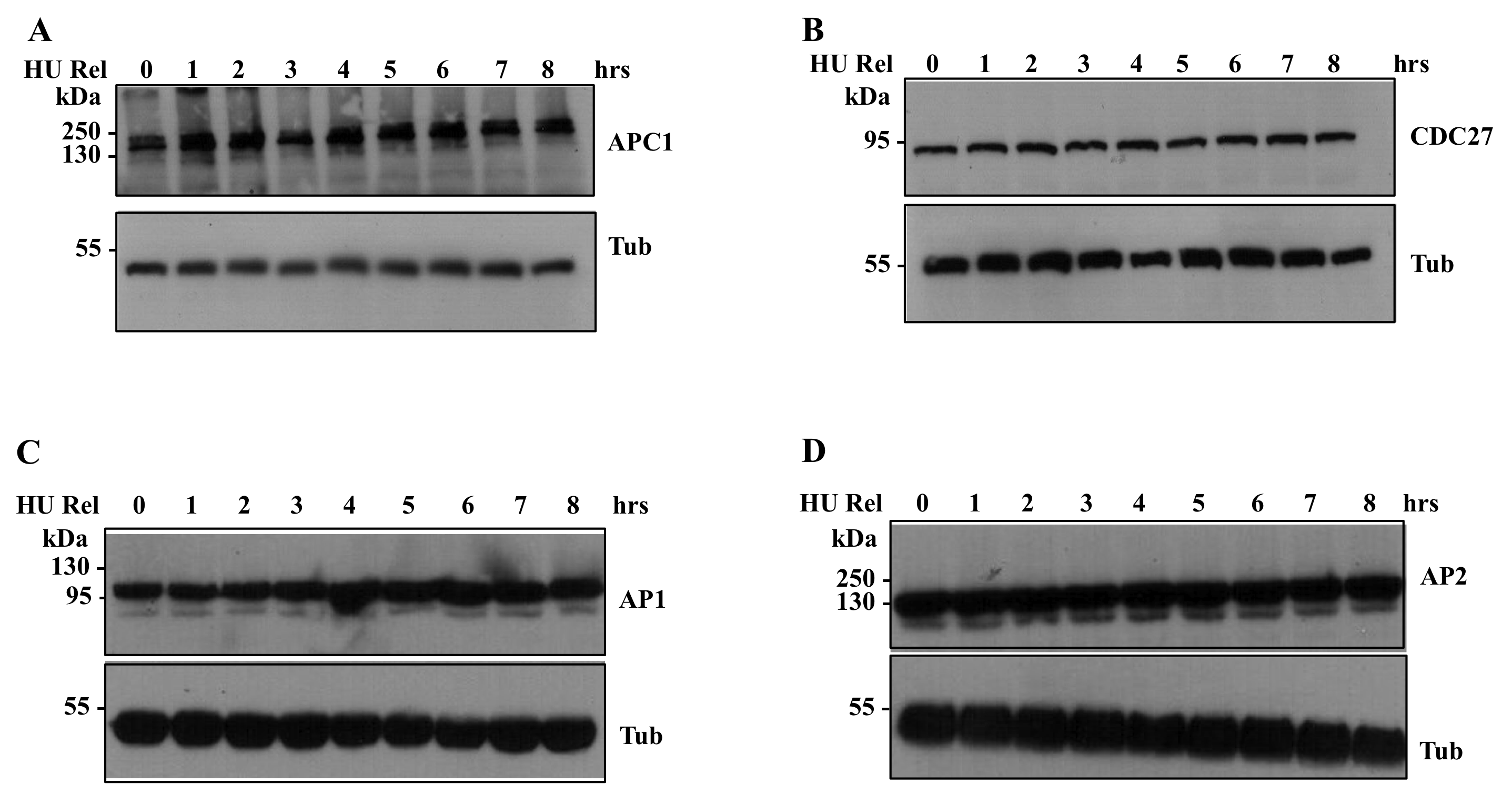

Supplement: Figure S2 — Time courses of expression of APC1, CDC27, AP1 and AP2 in synchronized T. brucei growth. Cells expressing endogenous PTP fusion proteins of (A) APC1; (B) CDC27; (C) AP1 and (D) AP2 were arrested in late S-phase after 16 hr treatment with 0.3 mM hydroxyurea. Samples of the released cells were taken every hour and their lysates monitored for the expression of individual fusion proteins by immunoblotting using the HPC4 antibody with anti-tubulin antibody used as loading controls. (TIF) [file pone.0059258.s002.tif]

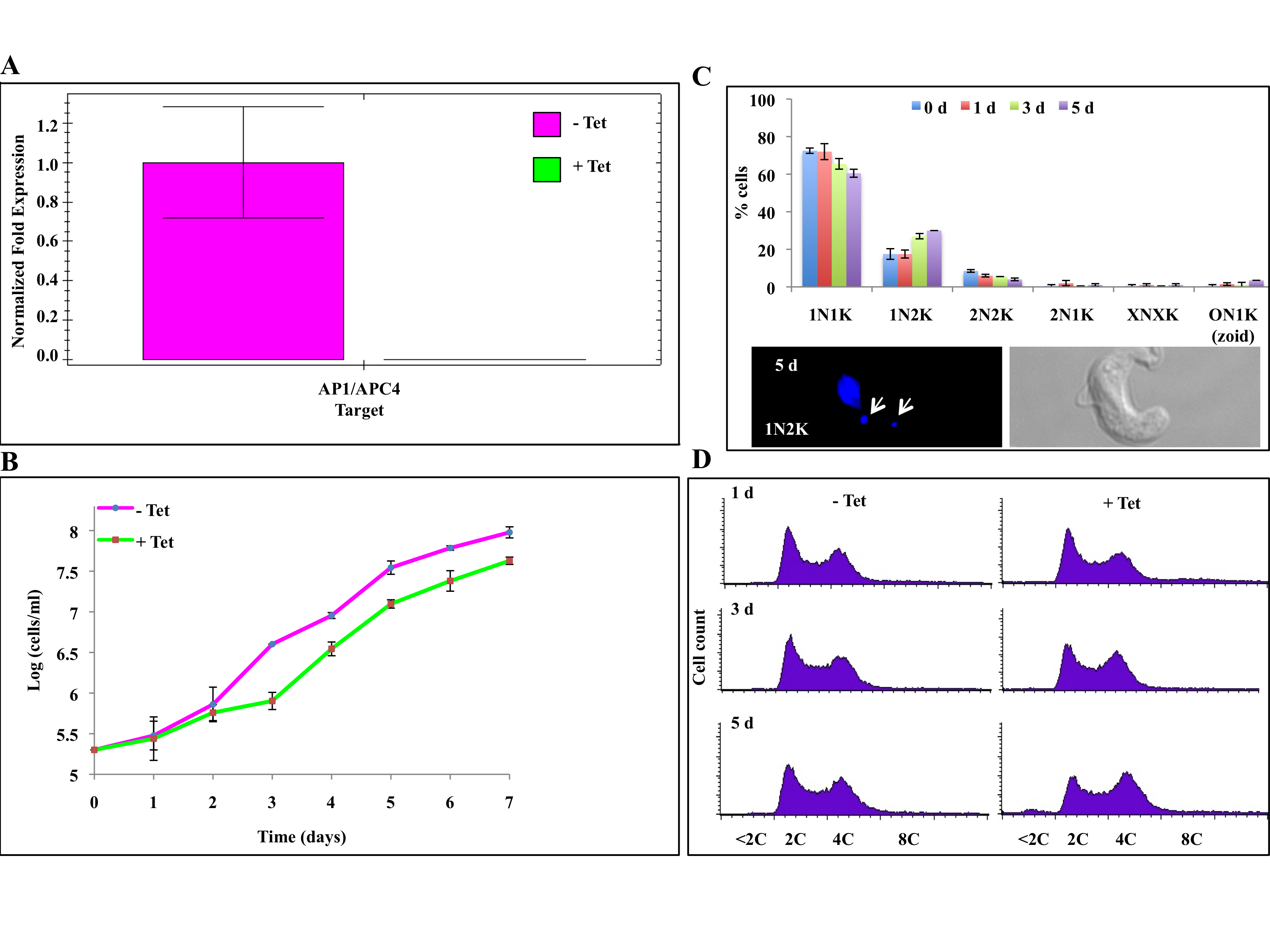

Supplement: Figure S3 — The RNAi knockdown of AP1/APC4. (A) qPCR assay of the level of AP1/APC4 mRNA 72 hrs after the induction of AP1/APC4 RNAi. (B) The rate of cell growth was monitored for 7 days after the RNAi induction. (C) N/K tabulations of the AP1/APC4-depleted cells on days 0, 1, 3 and 5 after RNAi induction. (D) Flow cytometric analysis of DNA contents in AP1/APC4-depleted cells. Little distinction was observed in the results from RNAi-induced and un-induced cells. (TIF) [file pone.0059258.s003.tif]

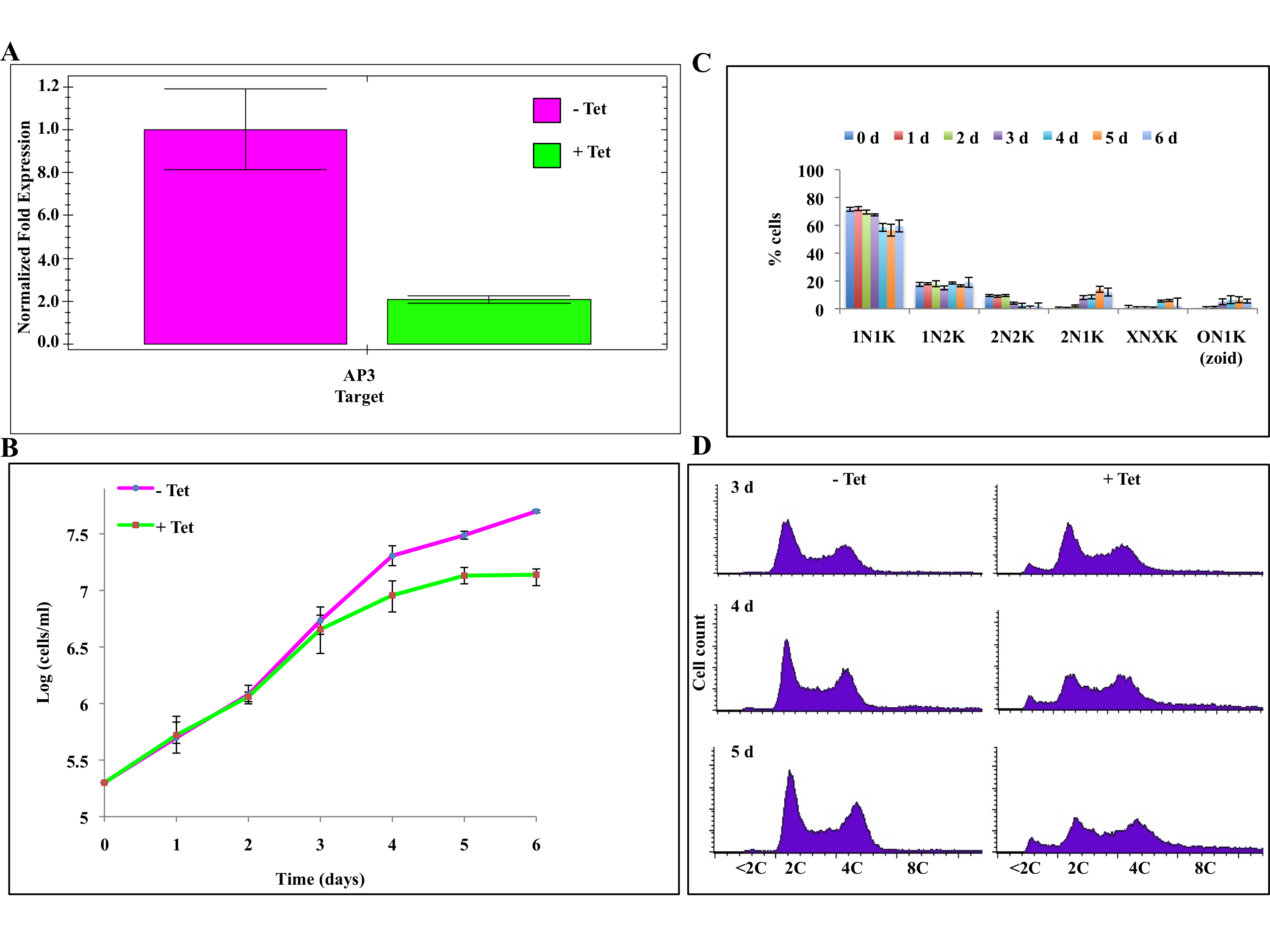

Supplement: Figure S4 — RNAi knockdown of AP3. Panels A, B, C and D are as described in Figure S3. (TIF) [file pone.0059258.s004.tif]

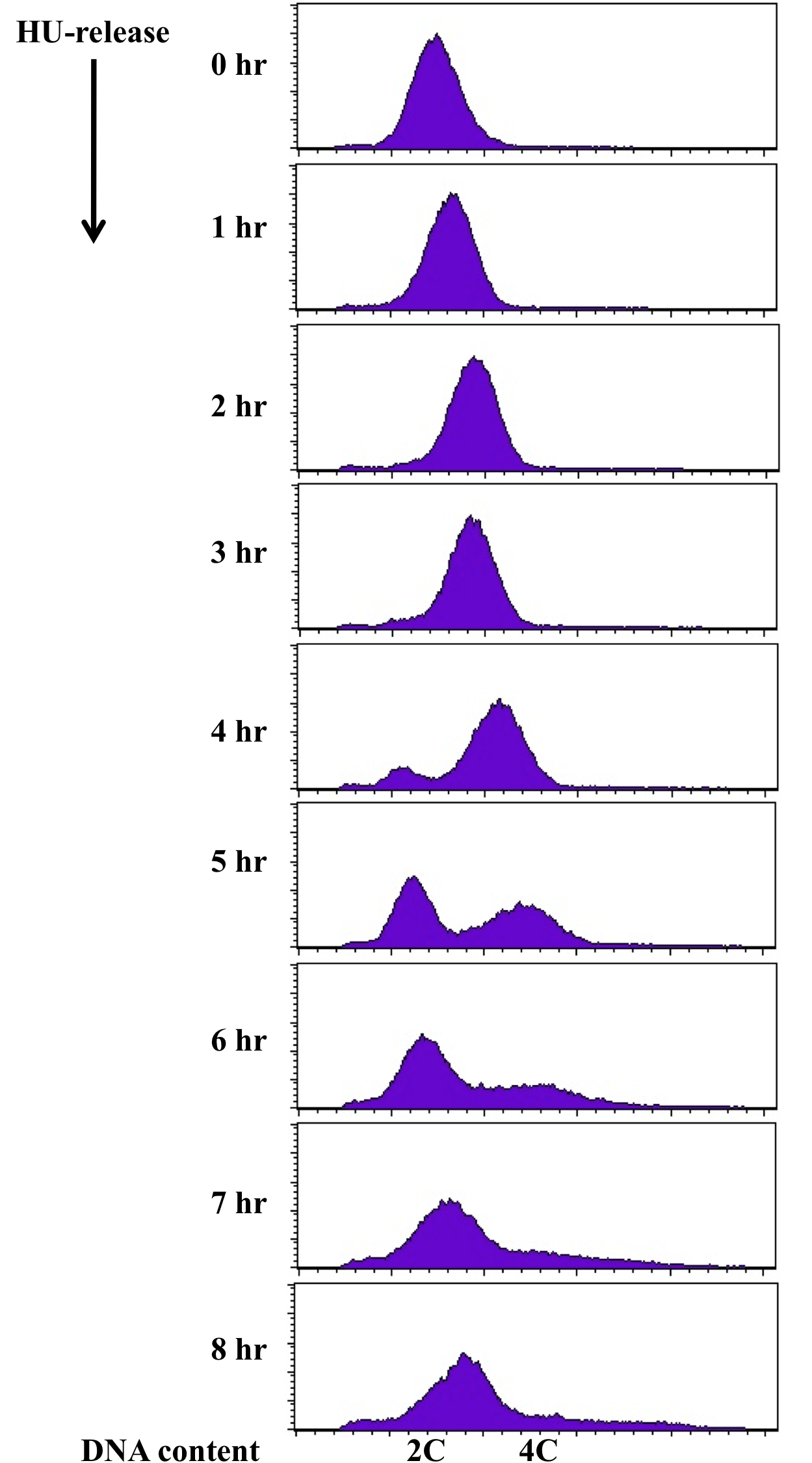

Supplement: Figure S5 — Synchronization of the cell cycle progression in T. brucei with hydroxyurea. Strain 29-13 procyclic T. brucei cells expressing CycB2/cyc6-3HA were treated with 0.3 mM hydroxyurea for 16 hours, washed twice in fresh medium and allowed to progress synchronously for 8 hours. The hourly cell samples were stained with propidium iodide, processed for flow cytometry and the FL2-A DNA peaks are presented. DNA contents (2C or 4C) were shown at the bottom. (TIF) [file pone.0059258.s005.tif]
